# Fitted peaks data of O^2−^–V^5+^ charge transfer bands and R/O data of Eu^3+^ doped Ca(VO_3_)_2_ and Ca_3_(VO_4_)_2_

**DOI:** 10.1016/j.dib.2018.01.087

**Published:** 2018-02-08

**Authors:** Ling Li, Yu Pan, Wenjun Wang, Zihao Wen, Xuanxi Leng, Qi Wang, Liqun Zhou, Haibing Xu, Qinghua Xia, Li Liu, Hongping Xiang, Xiaoguang Liu

**Affiliations:** a Hubei Collaborative Innovation Center for Advanced Organochemical Materials, Ministry-of-Education Key Laboratory for the Synthesis and Applications of Organic Functional Molecules, Inorganic chemistry, Hubei University, Wuhan 430062, China; b School of Materials Science and Engineering, Tongji University, 4800 Cao An Road, Shanghai 201804, China

## Abstract

*Data presented in this article are related to the research article entitled***O**^**2−**^**–V**^**5+**^**charge transfer band, chemical bond parameters and R/O of Eu**^**3+**^**doped Ca(VO**_**3**_**)**_**2**_**and Ca**_**3**_**(VO**_**4**_**)**_**2**_**: A comparable study[**ing Li, Yu Pan, Wenjun Wang, Zihao Wen, Xuanxi Leng, Qi Wang, Liqun Zhou, Haibing Xu, Qinghua Xia, Li Liu, Hongping Xian, Xiaoguang Liu]. The data present the fitting results of the broad excitation spectra of Ca(VO_3_)_2_:1%Eu and Ca_3_(VO_4_)_2_:1%Eu using the Gaussian model, the O/R values using the experimental PL emission results. The data compares the optimized cell parameters for Ca(VO_3_)_2_: 1%Eu and Ca_3_(VO_4_)_2_:1%Eu through the CASTEP geometry optimization with their initial cell parameters.

**Specifications Table**

**Table:** 

Subject area	*Chemistry*
More specific subject area	*Rare earth doped inorganic phosphors*
Type of data	*Table, figures*
How data was acquired	*PL(*time resolved fluorescence meter equipped with a 150 W Xe lamp as the excitation light source.)*, OriginPro 8*
Data format	*Raw, analyzed*
Experimental factors	*The positions and width of the fitted multiple peaks from a curve to fit Gaussian functions.*
Experimental features	*The data were determined directly through the experimental results.*
Data source location	*Department of Physics, Pukyong National University, Busan, Republic of Korea*
Data accessibility	*Data are presented in the article*
Related research article	L. Li, X. Liu, H.M. Noh, S.H. Park, J.H. Jeong, K.H. Kim, Key chemical parameters related to the width of the charge transfer band and the emission intensity of ^5^D_0_–^7^F_2_ in Eu^3+^ doped Ln_2_O_3_, J. Alloys Compd., 620 (2015) 324-328.

**Value of the data**

1. The data present the fitting results of the broad excitation spectra of Ca(VO_3_)_2_:1%Eu and Ca_3_(VO_4_)_2_:1%Eu using the Gaussian model.
2. The data provides the O/R values using the experimental PL emission results.
3. The data compares the optimized cell parameters for Ca(VO_3_)_2_: 1%Eu and Ca_3_(VO_4_)_2_:1%Eu through the CASTEP geometry optimization with their initial cell parameters.

## 1 Data

**Fig. 1:**
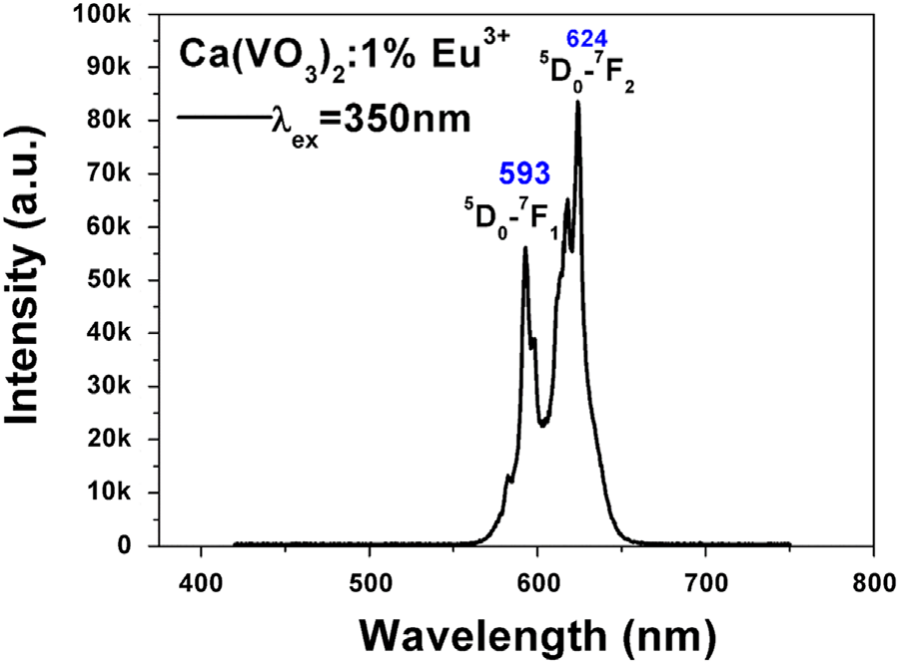
The typical PL spectrum of Ca(VO_3_)_2_:1%Eu when the excitation peak is 350 nm.

**Fig. 2:**
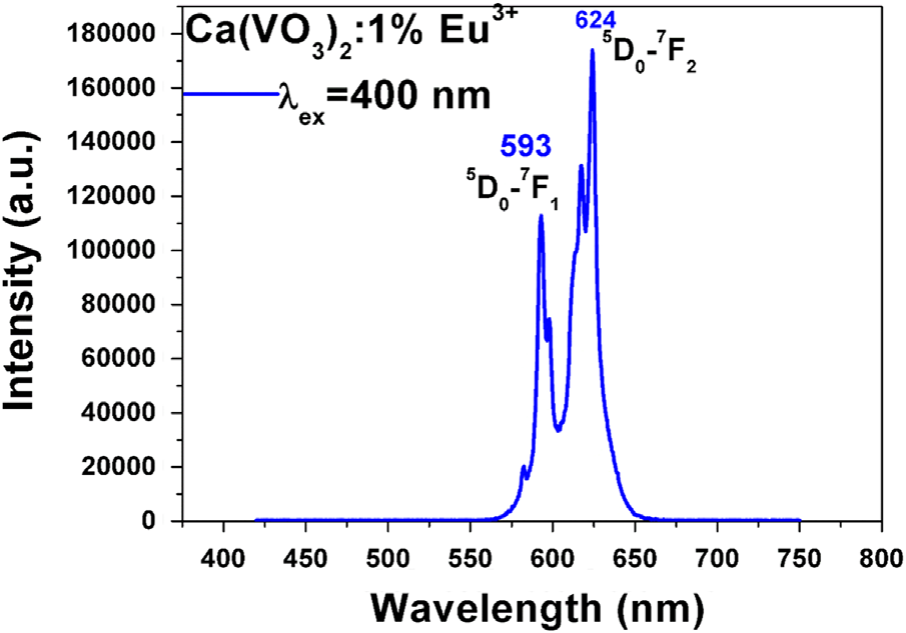
The typical PL spectrum of Ca(VO_3_)_2_:1%Eu when the excitation peak is 400 nm.

**Fig. 3:**
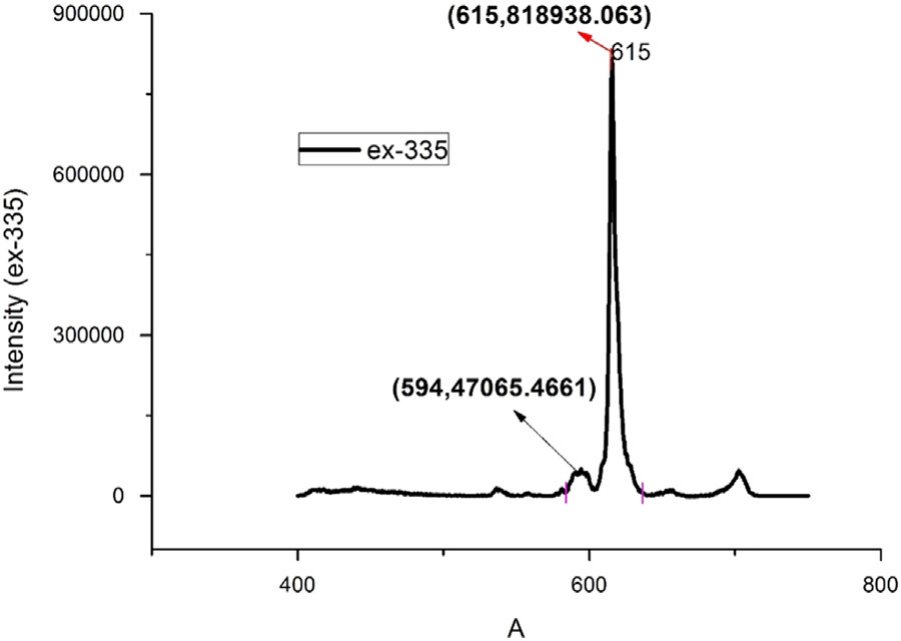
The typical PL spectrum of Ca_3_(VO_4_)_2_:1%Eu when the excitation peak is 335 nm.

**Fig. 4:**
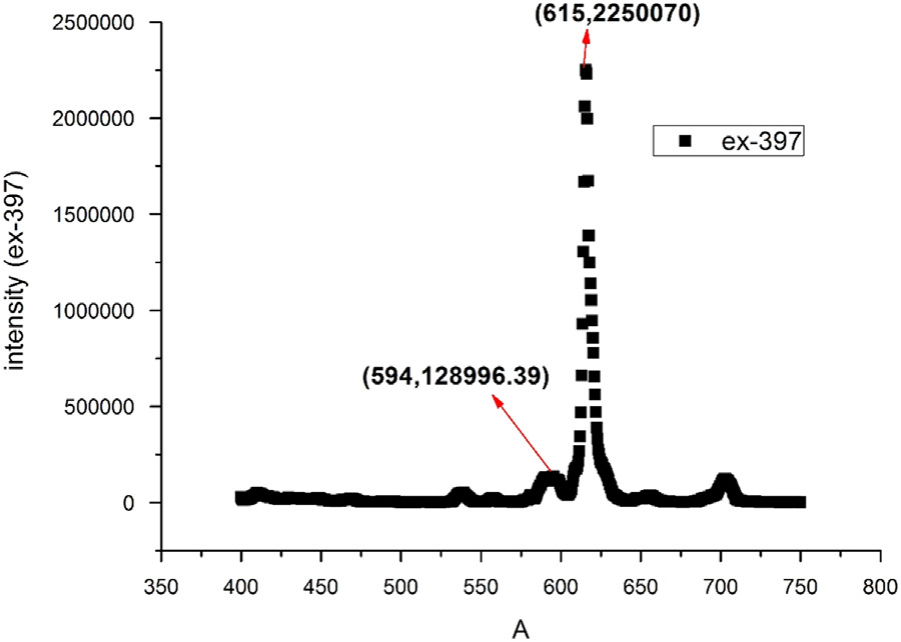
The typical PL spectrum of Ca_3_(VO_4_)_2_:1%Eu when the excitation peak is 397 nm.

The intensity ratios (R/O) of the ^5^D_0_→^7^F_2_ transition (red, its intensity is labeled using “R”) to the ^5^D_0_→^7^F_1_ transition (orange, its intensity is labeled using “O”) measuring the symmetry of Eu^3+^ are calculated [1], [2]. Fig. 1, Fig. 2 give the PL spectra when the excitation of the Ca(VO_3_)_2_: 1%Eu powder is at 350 and 400 nm, respectively. Fig. 3, Fig. 4 give the PL when the excitation of the Ca_3_(VO_4_)_2_: 1%Eu powder is at 335 and 397 nm, respectively. The R/O value is calculated using the experimental results, and the detail data are presented.

In the typical PL spectrum of Ca(VO_3_)_2_:1%Eu when the excitation peak is 350 nm, ID05→F27=83120(whenλem=624nm)andID05→F17=55455(whenλem=593nm)

So, ID05→F27ID05→F17=1.5

In the typical PL spectrum of Ca(VO_3_)_2_:1%Eu when the excitation peak is 400 nm, ID05→F27=173956(whenλem=624nm)andID05→F17=112777(whenλem=593nm)

So, ID05→F27ID05→F17=1.5.

In the typical PL spectrum of Ca_3_(VO_4_)_2_:1%Eu when the excitation peak is 335 nm, ID05→F27=818938.063(whenλem=615.5nm)andID05→F17=47065.4661(whenλem=594nm)

So, ID05→F27ID05→F17=17.4

In the typical PL spectrum of Ca_3_(VO_4_)_2_:1%Eu when the excitation peak is 397 nm, ID05→F27=2250070(whenλem=615nm)andID05→F17=128996.39(whenλem=594nm)

So, ID05→F27ID05→F17=17.4.

**Fig. 5:**
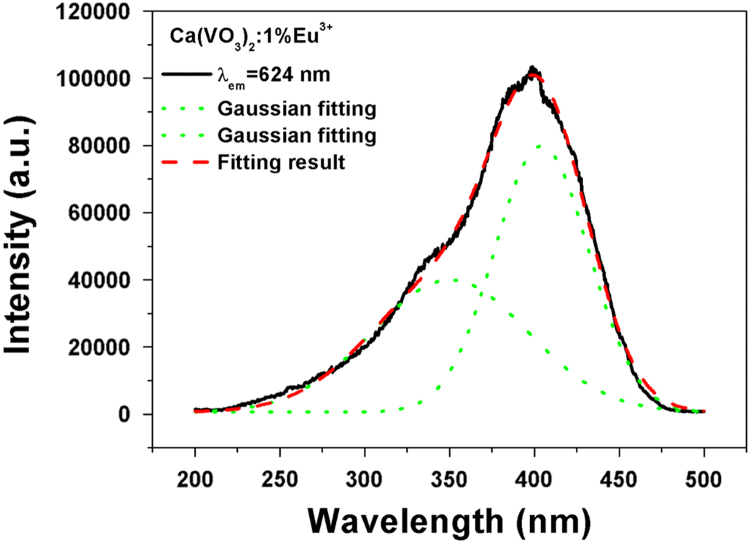
Typical photoluminescence excitation (PLE) spectra spectrum of Ca(VO_3_)_2_:1%Eu(λ_em_ = 624 nm). It was fitted by Gaussian functions shown in green dot curves. (For interpretation of the references to color in this figure legend, the reader is referred to the web version of this article.)

**Fig. 6:**
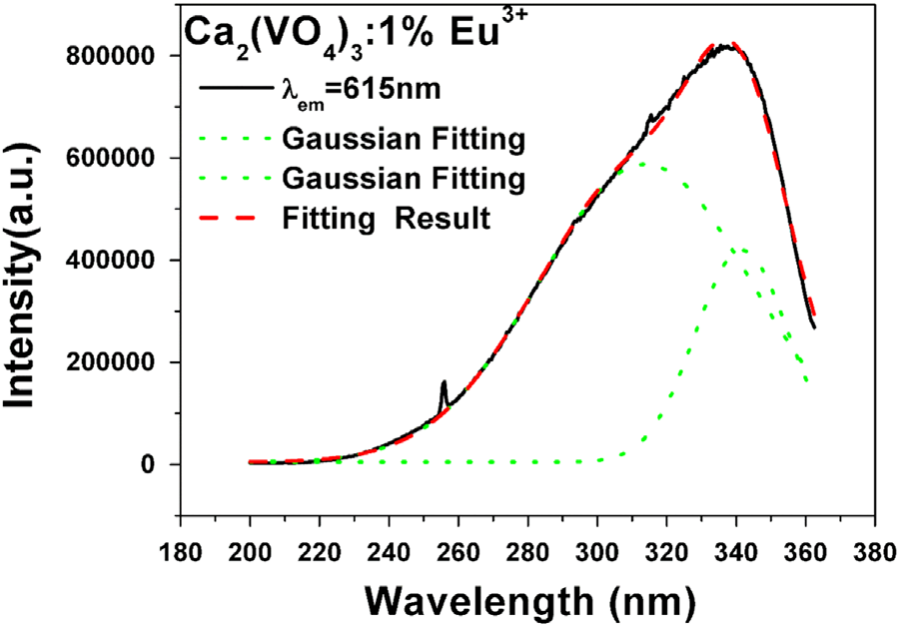
Typical photoluminescence excitation (PLE) spectra spectrum of Ca_3_(VO_4_)_2_:1%Eu. It was fitted by Gaussian functions shown in green dot curves. (For interpretation of the references to color in this figure legend, the reader is referred to the web version of this article.)

Fig. 5, Fig. 6 show the typical photoluminescence excitation (PLE) spectra spectrum of Ca(VO_3_)_2_:1%Eu and Ca_3_(VO_4_)_2_:1%Eu. They are fitted by Gaussian functions shown in green dot curves. This process is fitted using the Fit-multi peaks of Originpro 8.0 software. In the fitting dialog, the peak type was set as Gaussian and the number of peaks was set as 2. The black lines are the experimental results and red dotted lines indicate excitation spectra fitted with two Gaussian curves (green dotted lines) corresponding to the two excitation bands (350 and 404 nm for Ca(VO_3_)_2_:1%Eu; 313 and 341 nm for Ca_3_(VO_4_)_2_:1%Eu).

The data compares the optimized cell parameters for Ca(VO_3_)_2_: 1%Eu and Ca_3_(VO_4_)_2_:1%Eu through the CASTEP geometry optimization with their initial cell parameters. Table 1 presents Initial and optimized cell parameters for Ca(VO_3_)_2_: 1%Eu and Ca_3_(VO_4_)_2_:1%Eu.

**Table 1:** Initial and optimized cell parameters for Ca(VO_3_)_2_: 1%Eu and Ca_3_(VO_4_)_2_:1%Eu.

Crystal name	a(Å)	b(Å)	c(Å)	α(°)	β(°)	γ(°)
Ca(VO_3_)_2_:1%Eu^a^	10.0560	3.6730	7.0362	90	104.83	90
Ca(VO_3_)_2_:1%Eu^b^	10.6892	3.6582	7.2250	90	103.09	90
Ca_3_(VO_4_)_2_:1%Eu^a^	10.8141	10.8141	38.0235	90	90	120
Ca_3_(VO_4_)_2_:1%Eu^b^	11.3270	11.3270	42.0854	90	90	120

a It stands for the initial cell parameters of Ca(VO_3_)_2_: 1%Eu and Ca_3_(VO_4_)_2_:1%Eu.

b It stands for their optimized cell parameters through the CASTEP geometry optimization.
