# Supplementary material for: Fitted peaks data of O2−–V5+ charge transfer bands and R/O data of Eu3+ doped Ca(VO3)2 and Ca3(VO4)2
Source: Data Brief. 2018 Feb 8;17:1153–7. doi: 10.1016/j.dib.2018.01.087 (PMC5988486; doi:10.1016/j.dib.2018.01.087)
Supplement: Supplementary file 1 — Supplementary material [file mmc1.docx]

Conflict of Interest

The author declares as No conflicts of Interest.
